# 

**Published:** 1881-01-20

**Authors:** 


### INDEX TO AUTHORS.

BARRY A. L.—Burns .......... 36

BEALL, Wm. T.—A Case of Malposition of the Fœtus. sixty hours in Labor .......... 368

CALDWELL, T. H.—Operation for Strictured Hernia; patient 92 years old .......... 91

DURHAM A. F.—Gonorrhœa .......... 129

FOTHERGILL.—Ipecac in Dyspepsia .......... 156

GREENLEY. T. B.—Progressive Locomotor Ataxi .......... 131
Treatment of Pneumonia .......... 289

HENDREE, J.—Chemistry of Common Life.... 114
Raw Meat Juice in Cases of Inanition from Irritable Stomach .......... 158

HARDMAN, L. G.—Phagomania .......... 367

HOBBS, A. G., Professor of Diseases of Eye, Ear and Throat, in Southern Medical College, Atlanta, Ga.—Central Scotoma... 334

HOUSTON, Thomas.—A Case of Pleuritic Effusion .......... 203

KNOTT, J. S.—Unknown Eruptive Diseases,... 193

LALLERSTEDT, T. S.—Puerperal Convulsions .......... 169

LAW, J. H.—Emphysema and Bronchial Dilatation .......... 291

LOVING, A. B.—Rotheln .......... 251

MANN, Edward C.—The Nature, Pathology and Tr'm't of Dipsomania .......... 361, 401

MILLER, Charles H.—Epidemic Metastatic Parotidis .......... 252

McCONNELL, R. T.—Metastatic Prostatitis..... 353

PARHAM, E. H.—Hemorrhagic Fever successfully treated vithout Quinine .......... 48

POWELL, Prof. T. S. – Conversations upon the Physical and Mental Hygiene of Girlhood.......1, 41, 81, 121, 161, 201, 241, 281, 321

ROY, G. G.—Syphilitic Phthisis in the Negro.. 171

WAGNER, C. H.—Remarks on the Germ Theory of Diseases .......... 332
A Case of Facial Paralysis and Lumbago 369

WORD, Prof R. C.—Remarks upon the Treatment of pneumonia .......... 201
Clinical Lecture: Asthma — Vesicular Emphysema—Hay Fever .......... 442

WRIGHT, Thos. R.—Rupture of the Urethra with Extravasation of urine, etc .......... 175

### INDEX.

A

Abdominal surgery and listerism ... 409
Abnormally high temperature ... 23
Abortion, viburnum prunifolium in ... 348
Abortive and curative treatment of small pox 146
Absorption, gastric ... 351
Abstracts of papers read at the international medical congress ... 370
Acetic acid in small pox ... 423
Aconite in remittent fever ... 309
Action of bromide potassium ... 190
Acute articular rheumatism by subcutaneous injections of ergotine ... 69
Acute catarrh ... 114
Acute rheumatism ... 421
Administration of medicines, easy ... 301
After pains, chloral in ... 66
Albumen in the urine, new test for ... 470
Albuminuria, eucalyptol in ... 428
Albuminuria in pregnancy ... 234
Alcohol ... 469
Alcohol habits, coca in the opium and ... 185
Alcoholic mania in connection with acute disease, by J. B. Ayer ... 92
Alkalies in anæmia ... 24
Alopecia, syphilitic ... 422
Alstonia constricta ... 144
Amenorrhœa ... 35, 275
American medical association, catalogue of members of ... 117
American public health association ... 396
Ammonia, carbolate of ... 130
Ammonia, carbonate of ... 24
Anæmia, alkalies in ... 24
Anæmia of chlorosis ... 153
Anæsthesia in the larynx, new method of producing ... 426
Anæsthesia, morphia and chloroform combined ... 224
Anæsthetic, a new ... 302
Anæsthetic car ... 349
Anæsthetic method, an important modification of the ordinary ... 263
Anæsthetics in labor ... 104
Anæsthetics, minor ... 100
Anæsthetics, the limit of dangers in the use of 163
Animal heat ... 231
Anorexia, treatment of ... 354
Anthracæmia—wool sorters disease ... 186
Antidote for carbolic acid ... 154
Anti-emetic ... 393
Antiferment, a new ... 347
Antiseptic, a new ... 26
Antiseptic, a powerful ... 149
Antiseptic inhalation ... 434
Antiseptic treatment of carbuncle ... 146
Antiseptic treatment of enteric fever ... 353
Aphasia ... 219
Apparent death as a result of asphyxia ... 68
Aromatic tetrachloride of carbon ... 224
Arsenic in consumption ... 179
Arthritis ... 234
Artificial vaccine ... 145
Asphyxia, apparent death as a result of ... 68
Asphyxia from lack of air, interval between apparent and real death in ... 265
Aspiration of the gall bladder ... 381
Asthma ... 36, 76, 188, 236
Asthma, French treatment of ... 2[illegible]9
Asthma, lecture on, by Prof. Word ... 441
Asthma, treatment of ... 15[illegible]
Asthma with the induced current, treatm't of.. 105
Atomization in pulmonary hemorrhage, by Dr. Willard H. Morse ... 96
Atrophy of infants ... 80
Atropia and duboisia ... 350
Atropiæ sulphate, hypodermic injection of ... 222
Atropia for hysteria ... 189
Atropia for vomiting in pregnancy ... 189
Atropurpureus euonymus ... 346
Autopsy in the President's case ... 358
Azotite of ethyl ... 189

B

Bacteria fallacy illustrated ... 305
Balloons in submarine work ... 391
Bark in substance, value of, by Dr. William 9
A. Dayton ... 296
Beat production, repression of ... 474
Bees, providence of ... 112, 311
Belladonna as an antidote to opium poisoning 458
Belladonna in dysentery ... 316
Benzoate of sodium in acute rheumatism ... 269
Benzoic acid, physiological and therapeutical action of ... 266
Beverages, effervescent ... 75
Bicarbonate of soda in tonsilitis ... 429
Bichromate of potash ... 316
Bimeconate of morphia ... 187
Bill to regulate practice of medicine in Ga ... 357
Bismuth hair dye ... 74
Black haw ... 412
Bladder, irritable ... 233
Blood desiccated defibrinated ... 23
Blood, microscopic examination of ... 311
Board of health and the international sanitary conference of 1881, the national ... 267
Book notices ... 39, 79, 159, 199, 238, 319, 398, 438, 478
Boracic acid, caroba and iodoform in syphilis. 352
Boracic acid for cholera ... 225
Boracic acid in surgery ... 26
Boron, acid of ... 113
Bread poultice, common ... 389
Break-bone fever ... 15
Breast, potassium bromide in inflamed ... 427
Bright's disease, fuchsine in ... 109
Bromide of ammonium, therapeutic indications of ... 243
Bromide potassium, action of ... 190
Bromides, quinine with the ... 26
Bronchitis ... 76
Bronzing liquid ... 312
Burns ... 36, 51, 428

C

Cables and their life, transatlantic ... 311
Cables, submarine ... 191
Calcium, salicylate of ... 27
Camphor and chloral hydrate ... 24, 415
Cancer by chian turpentine, treatment of ... 26
Cancer cure ... 35
Cancer of the pancreas, primary ... 316
Cancer of the tongue ... 267
Cancrum oris ... 451
Carbolate of ammonia ... 24, 180
Carbolic acid, antidote for ... 154
Carbolic acid, radical treatm't of hydrocele by 384
Carbuncle, antiseptic treatment of ... 146
Caroba, iodoform and boracic acid in syphilis 35[illegible]
Cascara sagrada or rhamnus purshiana ... 230
Catalogue of members of the American Medical Association ... 117
Catarrh, acute ... 114
Catarrh and dysp'sia, by Dr. A. P. Whitehead. 257
Catarrh, chronic nasal ... 136
Catarrh, dry ... 393
Catarrh, nasal ... 30, 420
Catarrh of the nasal passage, almost a specific for ... 143
Caustic powder, Esmarch's ... 73, 235
Caustic, sulphate of zinc as a ... 315
Cement, aquarium ... 478

Cerebro-spinal meningitis.... 260
Chalk mixture for infantile diarrhœa.... 316
Chemistry of common life.... 114
Chian turpentine, treatment of cancer by.... 26
Chilblains, remedies for.... 76
Childhood, chorea of.... 429
Children, for the dysenteric diarrhœa in.... 314
Children, summer diarrhœa of.... 273
Children, summer dysentery and diarrhœa of teething.... 213, 274. 299
Children, summer dysentery of.... 299
Chloral cure for toothache.... 70
Chloral Hydrate, uses of.... 454
Chloral in after-pains.... 66
Chloral in dysentery.... 314, 306
Chlorate of potash, dangers of.... 229
Chlorate potassa.... 283, 316
Chloral hydrate for toothache.... 230
Chloride of iron, tincture of .... 432
Chloroform in diphtheria.... 270
Chloroform narcosis, to terminate.... 150
Chlorosis, for the anæmia of.... 153
Cholera, boracic acid for.... 225
Choleraic diarhœa by hypodermic injection of morphia, treatment of.... 76
Cholera infantum.... 110. 310, 316
Chorea of childhood.... 429
Chronic cystitis .... 233
Chronic dysentery cured by large dose of ipecacuahna, by Dr. W. S. Whitehead .... 213
Chronic rheumatism.... 29
Chronic ulcers.... 395
Clinical lecture, by Prof. Robert C. Word,.... 411
Coal tar, quinia from.... 190
Coca in alcohol habits .... 185
Coca in the opium habit.... 30
Cod-liver oil, administration of.... 235
Cod-liver oil, emulsions of.... 275
Coffee, hypodermic injection of,.... 224
Cold fire.... 472
Capsulotomy.... 469
Coca, a cure for morphinism.... 468
Cold weather and health.... 192
Colic, sodium phosphate in.... 227 462
Cologne, gold medal.... 194
Color of ozone, the.... 71
Cold, catching.... 466
Concentrated solution of quinia.... 217
Conjunctivitis, a case of phlyctenular.... 142
Constipation.... 274
Constipation, for habitual.... 475
Constipation, psyllium seed in .... 229
Consultations.... 77
Consumption, arsenic in.... 179
Copaiva in sciatica .... 156
Conversations upon the physical and mental hygiene of girlhood, by Prof. T. S. Powell, .... 1, 41, 81, 121, 161, 201, 241. 281, 321
Corns, preparation for.... 236
Corns, remedy for.... 155
Corpulency reduced by diet.... 189
Cotton goods uninflamable, rendering.... 392
Cotton-seed oil.... 431
Croup, diphtheritic.... 373
Croup, membranous.... 190
Croup treated by passing catheters into the trachœa by the mouth.... 337
Cystitis.... 154, 275

D

Dabel's purgative tincture .... 194
Damiana.... 179
Danger of uterine manipulations and operations .... 65
Dangers of tents.... 389
Death in asphyxia from lack of air, interval between apparent and real .... 265
Death of Dr. Tanner, reported.... 388
Death, painlessness of.... 392
Death smell .... 270
Delirium in acute rheumatism from salicylate of soda.... 149
Delirium tremens.... 68
Dentaphone, value of.... 272
Dentistry, a triumph of.... 280
Dermic dressing in variola.... 109
Desiccated, defebrinated blood.... 23
Despotism in lunatic asylums.... 147
Destruction of the membrana tympani, and application of the Toynbee disc, by Dr. A. S. Core.... 214
Diabetes mellitus, ergot in.... 27
Diagnosis of pregnancy.... 340
Diagnostic point in diseases, the tongue as a... 141
Diarrhœa and dysentery.... 313
Diarrhœa, chronic dysentery and .... 24
Diarrhœa, improved chalked mixture for infantile.... 316
Diarrhœa in children, for dysenteric.... 314
Diarrhœa infantile.... 307
Diarrhœal disorders of infancy.... 473
Diarrhœa, nervous.... 425
Diarrhœa of children, summer .... 273
Diarrhœa, salicylate in serous.... 273
Digestive agents in inflammatory rheumatism, by Dr. S. V. Swentningen.... 52
Digitalis.... 315
Digitalis in scarlet fever.... 187
Dilatation, emphysema and bronchial.... 291
Dinner pill, for a .... 390
Diphtheria.... 70, 73, 148, 194, 310
Diphtheria by tartaric acid, treatment of. .... 33
Diphtheria, chloroform in.... 270
Diphtheria, iodine treatment of.... 144
Diphtheria, lemon juice in.... 268
Diphtheria, notes on the treatment of.... 149
Diphtheria, pilocarpin in.... 110
Diphtheria, special affection following.... 114
Diphtheria, tartaric acid in.... 33, 235
Diphtheria, treatment of.... 276
Dipsomania, by Dr. Edward Mann.... 361, 401
Dyspnœa, iodide of potassium in cardiac .... 306
Dyspnœa, the use of quebracho in.... 380
Doctors of medicine, Germany ha 8.400.... 225
Dogwood, Jamaica .... 25
Dover's powder, improved.... 236
Duboisia and atropia.... 350
Duration of pregnancy .... 249
Dysentery and diarrhœa .... 313
Dysentery and diarrhœa, chronic .... 24
Dysentery and diarrhœa of teething children. 274
Dysentery, belladonna in .... 316
Dysentery, chloral in.... 306, 314
Dysentery diarrhœa in children.... 314
Dysentery, excellent remedy for.... 314
Dysentery, for acute.... 314
Dysentery of children, summer.... 299
Dysentery revived, an old treatment of.... 269
Dysentery, subnitrate of bismuth in.... 314
Dysmenorrhœa.... 35, 113
Dysmenorrhœa, spasmodic.... 473
Dyspepsia, catarrh and .... 257
Dyspepsia, headache of.... 315
Dyspepsia, ipecac in .... 156
Dyspepsia, for.... 434
Dystocia, relief by episiotomy. by Dr. F. O. Nagle .... 255
Dwarfs and giants.... 431

E

Earache.... 267
Eczema.... 334
Eczema, Dr. Bulkley's ointment for.... 233
Eczema of the genitals.... 58
Eczema of the hand.... 385
Editorial notices.... 37, 77, 157, 195
Effervescent beverages .... 75
Electrical devices in fire engine houses.... 391
Electrical science.... 312
Electricity in diseases of the nervous system, constant galvanic current of, by Dr. E. C. Mann .... 292
Electricity of rubber.... 439
Electricity, treatment of lead colic by.... 24
Elixir, McMunn's.... 35
Emetic, a new use of an.... 178
Emphysema and bronchial dilatation, by Dr. Jas. H. Low.... 291
Emulsions of cod-liver oil.... 275
Epidemic metastatic parotiditis .... 262

Epigastric pressure in obstinate hiccough........ 305
Epilepsy, treatment of ........................... 424
Episiotomy, a case of dystocia treated by......... 255
Epithelioma of the tongue, inutility and dangers of medical treatment of..................... 336
Ergot............................................... 148
Ergot, fluid extract of............................ 23
Ergot in diabetes mellitus......................... 27
Ergotin in prolapsus ani........................... 275
Ergotin solution for hypodermic use................ 33
Ergotin, treatment of acute articular rheumatism by subcutaneous injections of.......... 69
Ergot in tuberculosis.............................. 388
Eruption, remedies for poison oak.................. 194
Eruptive diseases, unknown......................... 193
Erysipelas–case in practice........................ 219
Esmarch's caustic powder....................... 73, 235
Esophageal tube, to pass........................... 305
Ethylate of sodium in the treatment of nævus...... 184
Ethyle, azotite of................................. 189
Etiology of vertigo................................ 139
Eucalyptol in albuminuria.......................... 428
Eucalyptus globulus........................... 344, 425
Eugenol............................................ 352
Euonymus atropurpureus............................. 356
Excision of a portion of the stomach and duodenum.............................................. 218
Exhilarant mixture, Luton's........................ 394
Expectorant, stimulating .......................... 114
Experiments with the inhalation of sulphuric ether.............................................. 138
Expert testimony in Toledo......................... 182
Eye, effect of masturbation on..................... 66

F

Facts from the Small-Pox Hospital at Troy, N. Y., by Dr Clarkson C. Schuyler........... 205
Fever, antiseptic treatment of enteric.............. 353
Fever, break-bone .................................. 15
Fever by salicylate of soda, treatm't of typhoid 423
Fever, digitalis in scarlet.......................... 187
Fever gastric remittent............................. 268
Fever hay........................................... 180
Fever, ice to the abdomen in........................ 310
Fever, intermittent and remittent................... 418
Fever, iodide of potassium in typhoid...... 274, 693
Fever, prevention of scarlet........................ 253
Fevers, aconite in remittent........................ 309
Fever, simple continued ............................ 153
Fever successfully treated without quinine, hemorrhagic......................................... 48
Fever, the land origin of the yellow................ 306
Fever, typhoid...................................... 185
Fever, typho-malarial............................... 374
Fire engine houses, new electrical devices in... 391
Fish bones in pharynx............................... 390
Fistula in ano, a new knife for..................... 21
Flatulence, acidity and pyrosis, glycerine in gastric.............................................. 352
Fluid extract of ergot.............................. 23
Fluoric acid, cure of goiter by................. 310, 395
Fluxes, dietetic treatment of intestinal........... 469
Fœtal to maternal circulation, relation of......... 351
Fomentations, a ready method for preparing.. 297
Food for infants & invalids, peptonized milk 75
Foot powder, salicylic acid as a.................... 74
Forearm, fracture of the............................ 221
Forests are going, where our........................ 31
Fracture-apparatus, random notes on simplicity of.......................................... 9
Fracture of the forearm............................. 221
Frost-bite, remedy for.............................. 154
Fuchsine in Bright's disease......................... 109
Furniture polish .................................... 74

G

Gall-blader, aspiration of the....................... 381
Gangrene of the mouth, noma or...................... 348
Gastric absorption.................................. 351
Gastric remittent fever............................. 268
Gastri[illegible]................................... 114
Gastrotomy, by Dr. L. L. Staton .................... 17
Gastrotomy for intussusception, a successful operation of....................................... 460

Gelseminum.......................................... 316
Genitals, eczema of the............................. 58
Germs of disease in water........................... 152
Germ theory of diseases, remarks on, by Dr. C. H. Wagner.......................................... 332
Giants and dwarfs .................................. 431
Gleet............................................... 156
Glycerine and its uses, aromatized.................. 386
Glycerine in gastric flatulence, acidity, etc....... 352
Goiter by fluoric acid, cure of................ 310, 395
Gonorrhœa........................................... 390
Gonorrhœa, by Dr. A. F. Durham...................... 129
Gonorrhœa, case of.................................. 476
Gonorrhœa, for...................................... 435
Gonorrhœa, on the treatment of...................... 345
Gonorrhœa, rhus aromatica in........................ 354
Grafting the tomato on the potato................... 471
Grass grow, to hear ................................ 431
Green vomit, nature of.............................. 258
Gui eau is he insane ............................... 417
Gynæcology, iodoform in............................. 57

H

Hair-dye, bismuth .................................. 78
Hair-dye with one preparation ...................... 74
Hand, eczema of the................................. 384
Hay fever........................................... 180
Hay fever, treatment of............................. 313
Headache ...................................... 475, 476
Headache of dyspepsia............................... 315
Headache, remedy for................................ 250
Health, cold weather and............................ 192
Healthiest cities................................... 414
Heartburn, treatment of indigestion and............. 140
Heart's action when it has ceased to beat, restoring of......................................... 464
Heating, the Prall system of........................ 31
Hemorrhage, atomization in pulmonary................ 96
Hemorrhage during labor............................. 302
Hemorrhage, post-partum............................. 298
Hemorrhagic fever successfully treated without quinine........................................ 48
Hemorrhoids.................................... 149, 394
Hemorrhoids, inflamed............................... 474
Hemorrhoids, some points in the treatment of by Dr. William R.D. Blackwood.................. 267
Hepatomy for hydatids .............................. 344
Hernia, operation for strictured, by Dr. T. H. Caldwell ......................................... 91
Hernias of long standing, treatment of.............. 468
Heroic police....................................... 225
Hiccough, epigastric pressure in.................... 305
Hop bitters ........................................ 434
Hop bitters, English................................ 473
Human and animal variola ........................... 426
Human bone, successful transplantation of........ 351
Hydatids, hepatomy for.............................. 344
Hydrate, camphor and chloral .................. 24, 415
Hydrocele by injection of carbolic acid............. 384
Hydrocele, Defer's method of treatment of simple.............................................. 437
Hydrocele, treatment of............................. 476
Hydrocyanate of iron in the treatment of neuralgia, value of................................ 452
Hyoscyamin as a mydriatic, sulphate of.............. 143
Hypodermic injection of atropiæ sulphate—a specific for sciatica .......................... 222
Hypodermic injection of morphia, treatment of choleraic diarrhœa by ........................ 76
Hypodermic injection, purgatives by................. 390
Hypodermic injections of coffee..................... 224
Hypodermic injections of quinia..................... 341
Hypodermic injections of strychnia, case of prolapsus ani successfully treated by......... 56
Hypodermic use, ergotine solution for............... 33
Hysteria, atropia for............................... 189

I

Ice, poisonous...................................... 228
Ice to the abdomen in typhoid fever................. 310
Impotence, by Dr. Samuel W. Gross................... 13
Impotency........................................... 274
Indigestion and heartburn, treatment of............. 140

Inanition from irritable stomach. raw meat juice in cases of........ 153
Infancy, diarhœal disorders of........ 473
Infantile diarrhœa........ 307
Infantile diarrhœa, improved chalk mixture for........ 316
Infantile diarrhœa, potassium bromide in........ 145
Infants, atrophy of........ 30
Inhalation, a new method of........ 150
Inhalation, antiseptic........ 434
Inhalation of sulphuric ether, experiments with........ 138
Injection of carbolic acid, radical treatment of hydrocele by........ 384
Injections of ergotin, treatment of acute-articular rheumatism by subcutaneous........ 69
Insects, strength of........ 72
Intelligence, a new test of........ 192
Interesting case for practice........ 103
Intermittent and remittent fever........ 418
Intestine, incised wound of, by Dr. W. A. Newborn........ 413
Intestine removed, two yards of........ 384
Intra-uterine........ 25
Introduction of tracheal tubes by the mouth... 26
Intussusception, a successful operation of gastrotomy for........ 460
Iodide of potassium in cordiac dyspnœa........ 306
Iodide of potassium in typhoid fever........ 274, 393
Iodine as a specific in croupous pneumonia... 107
Iodine treatment of diphtheria........ 144
Iodoform, caroba and boracic acid in syphilis.. 352
Iodoform, how to cover odor of........ 313
Iodoform in gynæcology........ 57
Ipecac in dyspepsia........ 156
Ipecacuana, chronic dysentery cured by large dose of,........ 213
Ipecacuanha in jaundice........ 465
Iron, tenacity of........ 32
Irrigation and injection tube, double........ 308
Irritable bladder........ 233
Itch, treatment of........ 187

J

Jamaica dogwood........ 25, 309
Jaundice, ipecacuanha in........ 465

L

Labor, hemorrhage during........ 302
Labor, the best position for women in........ 461
Labor, the use of anæsthetics in........ 104
Lady pharmacists in Holland........ 478
Lamp chimneys, toughened........ 112
Lancet, the use of the........ 349
Larynx, a mechanical........ 333
Larynx, new method of producing anæsthesia in the........ 426
Laxative combined, Stomachic and........ 435
Lead colic by electricity, treatment of........ 24
Lemon juice in diphtheria........ 268
Lent's solution of quinia........ 36
Leprosy in the United States, prevalence of..... 108
Lightning, trees and........ 231
Light on the skin, effects of........ 430
Listerine........ 353
Listerism, abdominal surgery and........ 409
Lister's antiseptic treatm't in surgical wounds. 307
Lochial discharges, treatment in cases of........ 177
Locomotor ataxi, phosphide of zinc in,........ 303
Locomotor ataxi, progressive, by Dr. T. B. Greenley........ 131
Luminous paint........ 111
Lunatic asylums, despotism in........ 147
Luton's exhilarant mixture........ 394

M

Malarial attacks, recurrent of obstinate, by Dr. John H. Pool........ 415
Malaria, recent studies on the nature of........ 101
Malposition of the fœtus, sixty hours in labor, by Dr. William T. Beal........ 368
Male wet-nurses........ 427
Maltine, on the use of........ 477
Mania, puerperal........ 180
Man in America........ 152
Man may live, how long........ 271
Masturbation, affections of the eye caused by.. 211
Maternal circulation, relation of fœtal to........ 351
Mcmunn's elixir........ 35
Medical Board of Georgia abolished........ 356
Medical year 1881........ 22
Members of the American Medical Association, catalogue of........ 117
Membranous croup........ 190
Meningitis, cerebro spinal........ 260
Menthol........ 350
Metastatic parotiditis, by Dr. Chas. H. Miller... 252
Metastatic prostatitis........ 358
Meteorites........ 471
Microscopic examination of blood........ 311
Micturition, painful,........ 393
Milk, sugar of........ 178
Morbus coxarius treated by the physiological method........ 388
Morphia bimeconate of........ 187
Morphia, tartrate of........ 352
Morphia, treatment of choleraic diarrhœa by the hypodermic injection of........ 76
Morphinism, coca a cure for........ 468
Mortality on the globe........ 231
Mouth, noma or gangrene of the........ 348
Movement, a........ 471
Mydriatic, sulphate of hyoscyamin as a........ 143

N

Nævus, ethylate of sodium in the treatment of 184
Nævus, removal of........ 113
Nævus, treatment of........ 146
Narcosis, to terminate the chloroform........ 308
Narcotic, a new—pitchouri bidgeri........ 147
Nasal catarrh........ 30
Nasal catarrh, chronic........ 136
Nasal catarrh, treatment of........ 420
Nasal passage, almost a specific for catarrh of.. 143
National radiant fluid........ 111
Nervine........ 75
Nervous diarrhœa........ 425
Nervous system, the constant or galvanic current of electricity in diseases of the........ 209
Neuralgia........ 179
Neuralgia and rheumatism........ 273
Neuralgia and rheumatism, pepper mint in..... 274
Neuralgia of the testis, by Dr. Geo. Halsted Boyland........ 216
Neuralgia, syphilitic........ 234
Neuralgia, value of hydrocyanate of iron in the treatment of........ 452
Night sweats........ 354
Night sweats, cure for........ 225
Nipples, sore........ 154
Nitro-glycerine, some of the therapeutical uses of, by Dr. William H. Hammond........ 407
Nocturnal emissions........ 274
Noma or gangrene of the mouth........ 348

O

Obstetric aphorisms, by Dr, H. Webster Jones.. 416
Occurrence of [illegible] ls in men........ 71
Œsophagoscopes,........ 429
Oil, cotton-seed........ 431
Ointment for eczema, Dr. Bulkley's........ 233
Ointment of wormwood........ 236
Operative interference in gunshot wounds of peritoneum........ 228
Ophthalmology and otology, Lecture on........ 437
Opium and alcohol habits, coca in the........ 185
Opium habit........ 98, 350
Opium habit, coca in th........ 30
Opium poisoning, belladonna as an antidote to 458
Orchitis and inflamed breast, potassium bromide in........ 427
Organism in abdominal typhus........ 148
Os, ulceration of the........ 354
Otology........ 26
Otology and Ophthalmology, Lecture on........ 437
Otorrhœa........ 370
Ovariotomy........ 145

Oxide of boron.......... 112

Ozæma, treatment of,.......... 137

Ozone.......... 71

Ozone, the color of.......... 71

P

Painful micturition.......... 393
Painlessness of death.......... 392
Paint, luminous.......... 111
Pancreas, primary cancer of the.......... 316
Paralysis and lumbago, facial, by Dr. C. H. Wagner.......... 369
Parvules, Warner's.......... 33
Peppermint in neuralgia and rheumatism.......... 275
Peptonized milk as food for infants & invalids 75
Peritoneal surgery.......... 25
Peritoneum, operative interference in gun-shot wounds of.......... 228
Petroleum mass.......... 33
Petroleum on trees and bushes.......... 432
Phagomania, by Dr. L. G. Hardman.......... 367
Pharynx, fish bone in.......... 390
Phenol.......... 350
Phlyctenular conjunctivitis.......... 142
Phosphide of zinc in locomotor ataxi.......... 303
Photography, recent progress in.......... 151
Phthisis, treatment of.......... 63
Physiological method, morbus coxarius treated by the.......... 388
Pilocarpin.......... 27, 178
Pilocarpin in diphtheria.......... 110
Piratical medical journals.......... 317
Pitchouri bidgeri, a new narcotic.......... 147
Placenta, adherent.......... 304
Placenta, retained.......... 262
Plaster of Paris, tripolith a substitute for.......... 147
Plaster, thapsia.......... 268
Pleuretic effusion.......... 203
Pneumonia.......... 64
Pneumonia aborted with quinine.......... 465
Pneumonia, double, by Dr. E. P. Townsend.......... 133
Pneumonia fever, wet sheet in.......... 226
Pneumonia, iodine as a specific in croupous.......... 107
Pneumonia, remarks upon the treatment of, by Dr. R. C. Word.......... 201
Pneumonia, treatment of, by Dr. T. B. Greenley.......... 289
Poisoned soils, poisoned by vegetables from.......... 67
Poisoning by strichnia.......... 386
Poison oak eruptions, remedies for.......... 194
Poisonous ice.......... 228
Polish, furniture.......... 74
Population of the earth.......... 32
Post-partum hemorrhage.......... 298
Post-partum hemorrhage and its treatment, by Dr. S. P. Sackett,.......... 50
Post-partum hemorrhage, notes on, by Dr. E. E. Bodeman.......... 54
Potassium bromide in infantile diarrhœas.......... 145
Potassium bromide in orchitis an inflamed breast.......... 427
Potassium, chlorate of.......... 186
Poultice, common bread.......... 389
Practical journalism.......... 477
Prall system of heating.......... 31
Precocity a sign of inferiority.......... 232
Pregnancy, albuminuria in.......... 234
Pregnancy, atropia for vomiting in.......... 189
Pregnancy, duration of.......... 249
Pregnancy, vomiting in.......... 113
Prescriptions, two favorite.......... 36
Prevalence of leprosy in the United States.......... 108
Prizes worth striving for.......... 387
Progress in photography, recent.......... 151
Prolapse of the ovaries.......... 459
Prolapsus ani, ergotin in.......... 275
Prolapsus ani successfully treated by hypodermic injection of strychnia, by Dr. Leonard Weber.......... 56
Proportion of physicians to inhabitants in different countries, comparative.......... 470
Providence of bees.......... 112
Pruritus of pregnancy.......... 154
Pruritus vulvæ.......... 395
Psylium seed in constipation.......... 229
Puerperal convulsions, by Dr. T. S. Lallerstedt 169
Pulmonary hemorrhage, atomization in.......... 96
Purgative pills.......... 435
Purgatives by hypodermic injection.......... 390
Purgative tincture, Dabel's,.......... 194
Pyœmia, what is.......... 338
Pyrosis, glycerine in.......... 352

Q

Quebracho in dyspnœa, the use of.......... 380
Quicksilver.......... 472
Quillaia tooth wash.......... 233
Quinia, concentrated solution of.......... 217
Quinia from coal tar.......... 190
Quinia, hypodermic injection of.......... 341
Quinine, pneumonia aborted with.......... 465
Quinine, the use of.......... 433
Quinine with the bromide.......... 26
Quinoline, tartrate of.......... 420

R

Random notes on simplicity of fracture apparatus, by Dr. William H. Morse.......... 9
Ranula, treatment of.......... 382
Raw meat juice in cases of inanition from irritable stomach.......... 138
Rectal douche, hot.......... 470
Rectum, resection of.......... 150
Refrigeration and animal heat.......... 231
Remarks upon the treatment of pneumonia.......... 201
Removal of nævus.......... 113
Removal of spots, stains, etc.......... 34
Report, a false.......... 457
Report on yellow fever in U. S. S. Plymouth.......... 108
Reprehensible practice.......... 70
Resection of rectum.......... 150
Respirator, new.......... 23
Resurrection.......... 387
Rhamnus purshiana or cascara sagrada.......... 230
Rheumatism.......... 28
Rheumatism, acute.......... 421
Rheumatism, benzoate of sodium in acute.......... 269
Rheumatism by subcutaneous injections of ergotin, treatment of acute articular.......... 69
Rheumatism, digestive agents in infl'matory.. 52
Rheumatism, for chronic,.......... 29
Rheumatism from salicylate of soda, delirium in acute.......... 149
Rheumatism, and neuralgia.......... 273
Rheumatism, peppermint in.......... 274
Rheumatism, treatment for.......... 190
Rhus aromatica.......... 309
Rhus aromatica in gonorrhœa.......... 253
Rhus poisoning, sassafras oil in.......... 313
Rotheln, by Dr. A. B. Loving.......... 251
Rubber, electricity of.......... 432
Rumination, the process of.......... 271
Rupture of the urethra with extravasation of urine, by Dr. Thos. R. Wright.......... 172
Rupture, radical cure of.......... 310, 395
Rusted bolts, to remove.......... 312

S

Salicylate of calcium.......... 27
Salicylate of soda, delirium in acute rheumatism from.......... 149
Salicylate of soda, treatm t of typhoid fever by 423
Salicylates in serous diarrhœa.......... 273
Salicylic acid, abortive tr,tm't of small pox by 307
Salicylic acid as a foot powder.......... 74
Salicylic acid, a vehicle for.......... 73
Sand bag for the sick room.......... 233
Sanguis bovinus exsiccatus.......... 347
Sanitary Conference of 1881, the National Board of Health and the International.......... 267
Sassafras oil as a remedy for rhus poisoning.......... 213
Scabies, ointment for.......... 474
Scalp, skin grafting in loss of.......... 376
Scarlet fever, digitalis in.......... 187
Scarlet fever, preventive of, by Dr. J. B. Garrison.......... 253
Sciatica, a specific for.......... 222
Sciatica, copaiva in.......... 156

Sclerotic acid 351
Scotoma, central, by Prof. A. G. Hobbs 334
Skin, effect of light on the 430
Skin grafting in loss of scalp, by Dr. William L. Bradley 376
Sleep, how long should we 391
Small pox 180
Small pox, acetic acid in 423
Small pox by salicylic acid, abortive tr'tm't of 307
Small pox hospital, facts from the 205
Small-pox in Richmond 462
Small pox, on the abortive treatment of 146
Snake bite 181
Snakes 472
Snake stone of Ceylon 191
Soda mint 234
Sodium phosphate in habitual colic 227
Solution of quinia, Lent's 36
Somnolence, a case of prolonged 388
Sores, ointment for old 394
Sound into light, transforming 32
Spermatorrhœa, remedies for 155
Spleen, enlarged 387
Sponge tents 27
Spots, stains, etc., removal of 34
Spray, ether 178
Stain for mahogany cherry 472
Startin's mixture 194
Stimulating expectorant 114
Stomach and duodenum, excision of a portion of the 218
Stomach, for the 235
Stomachic and laxative combined 435
Stramonium 315
Strength of insects 72
Strictured hernia, operation for 91
Strychnia, case of prolapsus ani successfully treated by hypodermic injections of 56
Strychnia, poisoning of 386
Studies on the nature of malaria, recent 101
Styptic colloid 474
Submarine cables 191
Subnitrate of bismuth in dysentery 314
Sucking thumb 303
Sugar of milk 178
Suit for damages 22$
Sulphate of hyoscyamin as a mydriatic 143
Sulphate of zinc as a caustic 315
Sulphur and sugar 179
Sulphate ferri in suppuration 180
Sulphuric ether, experiments with the inhalation of 138
Sunstroke and its treatment 264
Surgery, boracic acid in 26
Surgery, peritoneal 25
Surgical wounds, Lister's antiseptic tr'tm't in 307
Syphilis by razor, propagation of 270
Syphilis, caroba, iodoform and boracic acid in 352
Syphilis, new treatment for 467
Syphilitic alopecia 422
Syphilitic neuralgia 234
Syphilitic phthisis in the negro, by Prof. G. G. Roy 175
Syphilitic ulceration of velum accompanied with a large perforation 106
Syphilization, protective 428

T

Tails in men, occurrence of 71
Tape-worm 395
Tartaric acid, treatment of diphtheria by. 32, 235
Tartrate of morphia 352
Tartrate of quinoline 430
Teeth, how to preserve the 223
Telegraphing without wires 232
Temperature, abnormally high 23
Tenacity of iron 32
Tents, dangers of 380
Tents, sponge 27
Testing of new drugs 183
Testis, neuralgia of the 216
Tetanus, traumatic 29
Test of intelligence, a new 192
Tetra chloride of carbon, aromatic 224
Thapsia plaster 268
Therapeutical indications of bromide of ammonium 343
Thermometry, medical 383
Thumb-sucking 303
Thymol 352
Tide, utilizing of the 31
Tonga 24
Tongue as a diagnostic point in diseases 141
Tongue, inutility and dangers of medical treatment of epithol oma of the 336
Tongue, cancer of the 267
Tonsilitis, bicarbonate of soda in 429
Tonsils, enlarged 304
Toothache 69
Toothache, chloral cure for 70
Toothache, chloral hydrate for 230
Tooth wash, quillaia 233
Toynbee disc, destruction of the membrana tympani and application of the 214
Tracheal tubes by the mouth, introduction of 26
Transforming sound into light 32
Traumatic tetanus 29
Trees and lightning 231
Trichinia spiralis 144
Trichlorophenol a new antiferment 347
Tripolith, a substitue for plaster of Paris 147
Tuberculosis by vaccination, transmission of 457
Tuberculosis, ergot in 388
Tully's powder 235
Typhoid fever 185
Typhoid fever by salicylate of soda. tr'tm't of 423
Typhoid fever, ice to the abdomen in 310
Typhoid fever, iodide of potassium in 274, 393
Typho malarial fever, by Dr. A. C, Osterman 374
Typhus, the organism in abdominal 148

U

Ulceration of the os 334
Ulceration of velum, syphilitic 106
Ulcers, chronic 395
Ulcers of the legs, treatment for chronic 467
Uninflamable, rendering cotton goods 392
Urethra with extravasation of urine, etc., rupture of the 172
Urine, new test for albumen in the 470
Uterine medication, intra 25
Uterine manipulations and operations, dangers of 65
Uticaria, acute 430
Utilizing of the tide 31

V

Vaccinate, how to 269
Vaccination, transmission of tuberculosis by 457
Vaccine, artificial 145
Vaccine views 62
Variola, dermic dressing in 109
Variola, human and animal 426
Vegetables from poison soils, poisoned by 67
Velum, syphilitic ulceration of 106
Venereal warts 276
Venesection 25
Vertigo, etiology of 139
Viburnum opulus 276
Viburnum prunifolium 220, 449
Viburnum prunifolium in threat'n'd abortion 348
Vomiting in pregnancy 113
Vomiting in pregnancy, atropia for 189
Vulvæ, pruritus 395

W

Walking, physiology of 72
Warts, venereal 276
Water, germs of disease in 152
Wet nurses, male 427
Wet sheet in pneumonic fever 226
Whooping cough 230
Wool-sorters disease, anthracæmia 186
Worms, for 474
Wormwood, ointment of 236

Y

Yellow fever in the U. S. S. Plymouth, report 108
Yellow fever, the land origin of 306

### EDITORIALS AND MISCELLANEOUS.

### *EDITORIAL NOTICES.*

*Wm. R. Warner & Co.*—See new advertisement of this staunch and reliable house in this number of our Journal.

SEE new insert or New Year's address of Messrs. Merrell, Thorpe & Lloyd, that excellent manufacturing house of Cincinnati.

*Schuman's Pharmacy.*—We invite attention to the advertisement in this issue of the above establishment. Mr. Schuman has held a high position in Atlanta as a careful and skillful pharmacist and druggist.

*A. A. Mellier.*—See the new advertisement of A. A. Mellier, wholesale druggist and dealer in surgical instruments, etc., St. Louis, Mo. This house comes highly recommended as an old and reliable establishment.

*A Working Friend.*—Dr. A. T. Park, of Georgia, has placed us under strong obligations by sending a club of *twenty-eight* names for the RECORD for the year 1881. The Doctor is an active and progressive man in the profession, and a gentleman of warm and generous impulses.

*Geo. J. Howard & Bro., Wholesale Druggists, Atlanta.*—We invite attention to the advertisement of the above firm. They are good men and have opened up a large and splendid assortment of goods in the heart of the city. The senior partner was formerly engaged in the drug business in this city and established a high reputation as a staunch, successful and reliable business man.

*Our Price-List.*—The price-list, as contained in our last, was incorrect, the proof having been unintentionally overlooked. It did injustice to the Atlanta market and to Messrs. Pemberton, Pullum & Co., that excellent drug firm, who have engaged to make the monthly revisions. The fault was not theirs, for they are careful and reliable business men, and sell goods at low figures. The present list will be found correct, and hereafter care will be taken that no such oversight shall again occur.

THE Leibig Laboratory and Chemical Works Co., (Agts. J. L. Berg & Co., New York) are supplying the profession with a number of very useful preparations—among which we mention with special favor their Cocoa Beef Tonic, as well adapted to low states of the system where a nutritive, stimulating and tonic agent is required. We have been informed that Dr. J. C. Le Hardy, of Savannah, Ga., reported the treatment of three cases of chronic dyspepsia with the Cocoa Beef Tonic, wherein the assimilation of food was very defective, and there was a general dimunition of weight. The results obtained in two weeks were very flattering—one patient gained five pounds in weight; another three pounds, and the third three pounds and a half. This is certainly a remarkable showing. In view of the late favorable reports as to the effects of Cocoa in counteracting the opium habit, it would seem that the above preparation would be admirably adapted to the low nervous condition to which opium eaters are so subject. Indeed, it might prove a superior form in which to administer the Cocoa for the relief of this unfortunate habit.

*A Reminder.*—We are constrained to remind our readers that the terms of this Journal are *cash in advance.* All the losses we have sustained have resulted from the waiving of this rule. In the desire to keep up and increase our list we have, in too many instances, granted time to medical brethren who, though promising, and no doubt intending to pay, have not done so, thus subjecting us to much loss. It is the universal experience of journalism, that the cash rule alone can succeed. It is better for both the reader and the publisher that this rule be observed, and we therefore request all our friends who have not remitted to do so at their earliest possible convenience.—MANAGING EDITOR.

WE invite attention to two advertisements, in this issue of our Journal, of Parke, Davis & Co., Detroit, Mich. This house is doing an immense work as manufacturing Chemists—especially in the line of new preparations. Their new therapeutical agents, introduced from abroad, are adding constantly to the armamentarium of the practitioner in the conflict with disease.. Their establishment is large, and among the most liberal, reliable and enterprising on the continent. It may truthfully be said that no country in the world is making such rapid strides in the department of Therapeutics as our own. Our manufacturing Chemists are, as a class, very able and enterprising, and vie with each other in the preparation of fine Chemicals and excellent compounds. Great attention is given to the purity of the drugs used, and to neatness and precision in putting up the goods. In all these particulars the house of Parke, Davis & Co., have attained a deservedly high and world-wide reputation, and their goods are in constant and increasing demand.

### *RENEWALS.*

The proposition made in our December number to enter all upon the list for 1881 who did not notify us to the contrary by the 20th of January seems to have met the favor of subscribers everywhere, *only one man* having ordered a discontinuance up to this date. While this is true of renewals, quite a large addition has been made to our list in new subscribers. This is very gratifying to the editors, as it shows that the Journal is not only holding its own, but is constantly growing in popularity with the profession.

### *WHY DON'T YOU WRITE?*

We again appeal to our readers to write. Every observing and practical man has probably learned something that others have not, and if so, it is his duty to give it to the profession. It is only in this way that we can hope to make progress in the profession. Those who do not choose to write for our Original department can write a practical note or send us a good formula. Or, if nothing else, can address a question through the Journal, to their professional brothers, who may thus be drawn out in the expression of their views. In this way facts may be elicited and our readers mutually profited.

### *RECEIPTED.*

[Receipts not acknowledged privately are entered here.]

1879. Drs. M Giesy, J L Hudson, M C Baldridge. 1880. Drs. W D Hunt, W N Ames, S G Nichols, A F Harris, J W Harris, N B Warner, W B Maxwell. McCallum, N G West, Jno O'Brien, T L Cook, G R Dozier, G W Bowling, W Coggin, H M Longino, L M Wood, A H Askew, J S Knott. 1881. Drs. W S Johnson, B F Rudisill, G M Patterson, E H Parrham, M K Harrison, J A Davidson, J Gerdine M J Luster, Samuel Pool, C F Rodgers, C F Yeager, R E Toombs, Z D Emerson. J T Cleveland, R H Edwards, T P Olliver, A V Ponder, 6 ms., P W Callahan, J D Harrell, W B King, D B Hamilton, J W Mitchell, J W Lee, J McMichael, to Jan. '82; W T Kendall J B Vandergriff, Nath Wofford, R J Milam, M D Miles, W B Allgood, T B Wright, W M Gay, C A Jordan, J C Treutham, H T Emerson, S J Ellis, G W Delaperriere, W E Courson, J H Redding, F H Peck, S P Clayton, D Long. B J Foster, I T Suggs, 6 ms. of '81; M B Pollard, C M Gibson, R A Shimpoch, '82; J R Harrison, A A Davidson, J C Moody, R S Forchand, C H Jones, J S Glenn, A T Park.

